# The role and clinical significance of tumor-associated macrophages in the epithelial–mesenchymal transition of lung cancer

**DOI:** 10.3389/fonc.2025.1571583

**Published:** 2025-04-15

**Authors:** Lei Liao, Ying-Xia Wang, Su-Su Fan, Ying-Yue Hu, Xue-Chang Wang, Xuan Zhang

**Affiliations:** ^1^ School of Pharmaceutical Sciences & Yunnan Key Laboratory of Pharmacology for Natural Products, Kunming Medical University, Kunming, China; ^2^ Department of Pathology, the First Affiliated Hospital of Kunming Medical University, Kunming, China; ^3^ Department of Pharmacy, Anning First People’s Hospital, Anning, China; ^4^ Yunnan College of Modern Biomedical Industry, Kunming, China

**Keywords:** tumor-associated macrophages, epithelial-mesenchymal transformation, lung cancer, tumor microenvironment, tumor therapy

## Abstract

Lung cancer remains the leading cause of cancer-related mortality worldwide. Tumor-associated macrophages (TAMs) and epithelial-mesenchymal transition (EMT) are key drivers of lung cancer metastasis and drug resistance. M2-polarized TAMs dominate the immunosuppressive tumor microenvironment (TME) and promote EMT through cytokines such as TGF-β, IL-6, and CCL2. Conversely, EMT-transformed tumor cells reinforce TAM recruitment and M2 polarization through immunomodulatory factors such as CCL2 and ZEB1, thereby establishing a bidirectional interplay that fuels tumor progression. Current evidence on this interaction remains fragmented, and a comprehensive review of the TAM-EMT regulatory network and its therapeutic implications is lacking. This review systematically integrates the bidirectional regulatory mechanisms between TAMs and EMT, highlighting their roles in lung cancer progression. It also summarizes emerging therapeutic strategies targeting TAM polarization and the EMT process, emphasizing their potential for clinical translation. This study fills the gap in systematic reviews on the interaction between TAMs and EMT, providing a comprehensive theoretical foundation for future research and the development of novel lung cancer therapies.

## Graphical Abstract

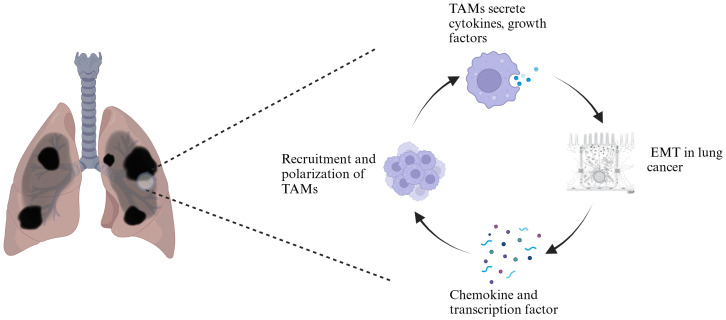

## 1 Introduction

Primary bronchogenic carcinoma, commonly referred to as lung cancer, is the most lethal malignancy worldwide and the second most common cancer in terms of incidence (1). Lung cancer can be classified into small cell carcinoma (SCLC) and nonsmall cell carcinoma (NSCLC) on the basis of the histological characteristics of the cancer cells. NSCLC is the most common subtype, accounting for 85–90% of all lung cancer types. NSCLC comprises several histological subtypes, including lung adenocarcinoma, squamous cell carcinoma, and large cell carcinoma (2). Currently, treatment options for lung cancer include surgical resection, chemotherapy, targeted therapy, and radiotherapy. Although progress has been made in early diagnosis and treatment, the overall prognosis for patients with lung cancer remains poor.

Macrophages that infiltrate or accumulate in the tumor microenvironment are defined as tumor-associated macrophages (TAMs) and represent the predominant infiltrating immune cells in solid tumors. They exhibit high plasticity, are capable of exhibiting protumor or antitumor functions in response to various signaling stimuli (3–5), and have multiple supportive and inhibitory effects on tumor growth, progression, and metastasis (6).

Epithelial–mesenchymal transition (EMT) refers to a highly programmed process in which epithelial cells lose their original phenotypic characteristics and acquire mesenchymal cell traits (7). During EMT, epithelial-derived tumor cells lose their epithelial characteristics and acquire mesenchymal traits, resulting in reduced intercellular adhesion, loss of cell polarity, and increased cell migration, invasion, and antiapoptotic capabilities. EMT plays a crucial role not only in embryonic development and tissue repair but also in tumor progression, participating in various tumor processes, including tumor initiation, stemness, migration, oncogenic progression, vascular infiltration, malignant metastasis, and resistance to therapy (8).

In recent years, TAMs and EMT have become hot topics in cancer research. Studies have shown that the interaction between TAMs and EMT is closely related to tumor progression (9–11). Although the association between TAMs and EMT has been preliminarily reported, the bidirectional regulatory mechanisms and clinical translational potential have yet to be systematically elucidated. This review aims to explore the role of TAMs in lung cancer EMT and their clinical significance. By systematically reviewing the relevant literature, this study analyzes the interaction between TAMs and EMT, reveals their roles in lung cancer progression, and discusses the clinical potential of targeting TAMs and the EMT process, aiming to provide a theoretical basis for future research and therapeutic strategies.

## 2 Tumor-associated macrophages

### 2.1 Sources and differentiation of TAMs

Pulmonary macrophages originate from two main lineages: tissue-resident macrophages (TRMs), which can locally self-renew independently of hematopoietic stem cell pathways in adults, and monocyte-derived macrophages (MDMs), which originate from adult hematopoietic stem cells and have a shorter lifespan and tend to accumulate at sites of inflammation (12). TAMs are key immune cells in the tumor microenvironment and are derived primarily from monocytes in the bloodstream; these cells migrate to the tumor microenvironment and differentiate, with a small portion also originating from TRMs (13). The differentiation and function of TAMs are regulated by various signals in the tumor microenvironment, including cytokines and chemokines secreted by tumor cells, stromal cells, and other immune cells. Existing studies indicate that TAMs can be classified into the classical activated M1 type and the alternatively activated M2 type (4, 14–17). M1-type TAMs induced by granulocyte-macrophage colony-stimulating factor (GM-CSF), interferon-γ (IFN-γ), tumor necrosis factor-α (TNF-α), lipopolysaccharides (LPS), or other pathogen-associated molecules are generally considered to exhibit pro-inflammatory and anti-tumor effects, expressing inflammatory factors such as interleukin (IL)-1β, IL-6, and TNF-α (14, 18–20); in contrast, M2-type TAMs induced by macrophage colony-stimulating factor (M-CSF), IL-4, IL-10, IL-13, transforming growth factor (TGF)-β, glucocorticoids, or immune complexes exhibit anti-inflammatory effects and promote tumor progression, expressing anti-inflammatory factors such as IL-10 and TGF-β (14, 19–21). With the advancement of research, M2-type TAMs can be further subdivided into M2a, involved in tissue fibrosis (induced by IL-4 or IL-13), M2b, which promote tumor progression (induced by immune complexes in conjunction with IL-1β or LPS), M2c, responsible for tissue remodeling (induced by IL-10, TGF-β, or glucocorticoids), and M2d, which promote angiogenesis (induced by IL-6, leukemia inhibitory factor (LIF), and adenosine) (6, 17, 18, 22) (**Table 1**).

**Table 1:** Types and functions of TAMs.

Types	Subtypes	Inducing Factors	Major Expressed Factors	Functions
M1-Type		GM-CSF, IFN-γ, TNF-α, LPS, etc.	Inflammatory factors such as IL-1β, IL-6, IL-12, IL-23, TNF-α, etc.	Pro-inflammatory and anti-tumor (6, 17).
M2-Type	M2a	IL-4、IL-13	CD206, IL-10, TGF-β, CCL17, CCL18, CCL22, etc.	Involved in tissue fibrosis and promoting wound healing (6, 18).
	M2b	Immune complexes with Toll-like receptor (TLR) ligands, IL-1β, LPS, etc.	TNFα, IL-1β, IL-6, and IL-10, etc.	Immune regulation and tumor progression (6, 18, 22).
	M2c	IL-10, TGF-β, glucocorticoids, etc.	IL-10, TGF-β, CCL16, and CCL18, etc.	Responsible for tissue remodeling and phagocytosing apoptotic cells (6, 18).
	M2d	IL-6, leukemia inhibitory factor (LIF), adenosine, etc.	VEGF, IL-10, and PD-1, etc.	Immune suppression and promotion of angiogenesis (6, 18).

### 2.2 TAMs and lung cancer

In the tumor microenvironment, the phenotype, distribution, and density of TAMs are closely related to patient prognosis (23–25). In lung cancer tissues, the number of M2-type TAMs is usually greater than that of M1-type TAMs (26–29). Compared with tumor nodules, M1 and M2 macrophages primarily infiltrate the tumor stroma, and a higher density of M1-type macrophages is often associated with better survival rates for patients (27, 30), whereas high infiltration of M2-type macrophages generally indicates poorer prognosis (28). Although M1-type macrophages are typically associated with tumor suppression, in the initial stages of tumorigenesis, they support the initiation of tumor development by producing reactive oxygen and nitrogen intermediates. These reactive oxygen and nitrogen intermediates induce DNA damage in proliferating cells and surrounding epithelial cells, thereby increasing the risk of tumor transformation (31). Therefore, during the initial stages of tumor development, M1-type TAMs are the predominant macrophages. However, in early lung cancer, M1-type and M2-type TAMs are not mutually exclusive; some M2-type TAMs also exhibit strong M1-type characteristics. Additionally, there is a strong correlation between the density of M1-type TAMs and the density of TRMs in tumors, which is associated with better survival (26).

During the wound healing process, the activities of M2-type macrophages can promote the restoration of tissue homeostasis. However, in the tumor microenvironment, the polarization of M2-type TAMs plays a crucial role in tumor progression (32). In the vast majority of solid tumors, including lung cancer, M2-type TAMs are positively correlated with tumor growth and metastasis. During the development of lung cancer, M2-type TAMs drive tumor cell proliferation, survival, epithelial-to-mesenchymal transition, and immune evasion by secreting a range of molecules, including growth factors, chemokines, cytokines, and matrix metalloproteinases (MMPs), thereby promoting the invasion and metastasis of tumor cells *in vivo (* 33–35). Among these, the expression of various molecules, such as JNK, HB-EGF, and Mincle, in M2-type macrophages promotes the growth of NSCLC cells (36, 37), whereas vascular endothelial growth factor (VEGF), platelet-derived growth factor (PDGF), IL-10, and exosomes containing miR-155 and miR-196a-5p are associated with lung cancer progression and metastasis (38). After lung cancer formation, TAMs in the tumor microenvironment induce M2 polarization through a series of complex molecular mechanisms, including the COX-2/PGE2/EP4 signaling axis, the AMPKα1/STING positive feedback regulatory pathways, the regulatory role of Zeb1 transcription factors, the involvement of CtBP1, the mediation by CCL2, and the secretion of circFARSA or the release of exosomes carrying PD-L1 (39–45). This leads to the gradual formation of a tumor microenvironment primarily characterized by M2 macrophages, which support and promote angiogenesis, tumor growth and survival, invasion and metastasis, and immune suppression, further driving the growth and metastasis of lung cancer.

## 3 Epithelial–mesenchymal transition

### 3.1 Overview of EMT

Epithelial–mesenchymal transition (EMT) is a complex and multifaceted process that plays a crucial role in embryonic development, tissue repair, and pathological conditions such as cancer and fibrosis. During the EMT process, epithelial cancer cells undergo a series of molecular changes, including downregulation of the epithelial marker E-cadherin and upregulation of the mesenchymal markers vimentin and N-cadherin, thereby acquiring the characteristics of mesenchymal cells. During this transition, epithelial cells lose their polarity and intercellular tight junctions, acquiring enhanced migratory and invasive properties that enable them to traverse the stroma and migrate to new locations. Cancer cells undergoing EMT secrete various cytokines to remodel the tumor immune microenvironment (46).

EMT results in three main phenotypes in different biological contexts (47, 48): (1) Embryonic EMT (also known as Type 1 EMT) primarily occurs during implantation, embryogenesis, and organ development and is driven by the evolutionary need to remodel and diversify tissues to achieve proper morphogenesis and produce functional organisms. This EMT is transient and unrelated to inflammation, fibrosis, or systemic dissemination. ② Regenerative EMT (Type 2 EMT) is closely related to wound healing, tissue regeneration, and organ fibrosis. In the context of injury, regenerative EMT promotes tissue repair by producing activated mesenchymal cells, particularly myofibroblasts, which generate excessive collagen-rich extracellular matrix (ECM). ③ Cancerous EMT (Type 3 EMT) occurs in the context of tumor growth and cancer progression, particularly in tumor cells that have previously undergone genetic and epigenetic changes. During this process, tumor cells convert to a mesenchymal phenotype, thereby acquiring invasive and metastatic capabilities, which are critical steps in cancer progression and metastasis.

### 3.2 EMT and lung cancer

In lung cancer, EMT is considered a key mechanism by which tumor cells acquire migratory and invasive abilities; this phenotype is commonly observed in primary squamous cell carcinoma (SCC) and lung adenocarcinoma (LUAD) and typically occurs early in the pathogenesis of lung cancer (49). The occurrence of EMT is closely associated with metastasis, drug resistance, immune evasion, and poor prognosis in patients with lung cancer (48). The expression levels of markers, such as E-cadherin, which maintains adhesive junctions, and vimentin, which is involved in cytoskeletal remodeling, are significantly related to the prognosis of non-small cell lung cancer (NSCLC) (50, 51). As a fundamental event in the EMT process, the loss of E-cadherin expression is a key step in tumor cell infiltration and progression (52). Studies have shown that conditional deletion of E-cadherin or the expression of dominant-negative E-cadherin leads to weakened intercellular adhesion among cancer cells and induces vascular invasion and tumor growth through the upregulation of VEGF-A and VEGF-C mediated by β-catenin, thereby inducing micrometastasis in lung adenocarcinoma (53). Furthermore, EMT is also associated with tumor stem cell properties, endowing tumor cells with enhanced migratory and invasive capabilities (54, 55).

On the other hand, several genes in lung cancer, such as Twist, Snail, and TGF-β1, have been confirmed to be associated with EMT, promoting the process of EMT in lung cancer by downregulating E-cadherin and upregulating Vimentin (56–58). Additionally, lung cancer cells can induce epithelial–mesenchymal transition (EMT) through various molecules and signaling pathways. In non-small cell lung cancer, the overexpression of FoxQ1, CTEN, and EDA fibronectin (EDAFN), as well as the activation of the RAGE receptor by advanced glycation end products, can induce EMT in lung cancer cells, promoting tumor progression (59–62). H.R. et al. (63) found that NSCLC cells can induce EMT and promote proliferation, migration, and invasion by downregulating SETBP, which activates the ERK1/2 pathway.

## 4 Interaction of TAMs with EMTs

### 4.1 How TAMs influence the EMT process in lung cancer cells

Tumor-associated macrophages (TAMs) exhibit a multilayered and profound regulatory effect on the epithelial–mesenchymal transition (EMT) process in the tumor microenvironment, and their quantity and activation state significantly correlate with EMT regulation. In various solid tumors, including non-small cell lung cancer, the overall quantity of TAMs and the high expression of specific markers (such as CD68 and CD163) are closely related to enhanced EMT characteristics in cancer cells (such as downregulation of E-cadherin and upregulation of vimentin), indicating that EMT features are more pronounced in tumor areas rich in TAMs (33, 64–66). Furthermore, during lung cancer progression, there is a significant increase in the number of TAMs and tumor-associated fibroblasts, along with a marked increase in the expression levels of EMT markers in tumor tissues (67).

**Figure 1:**
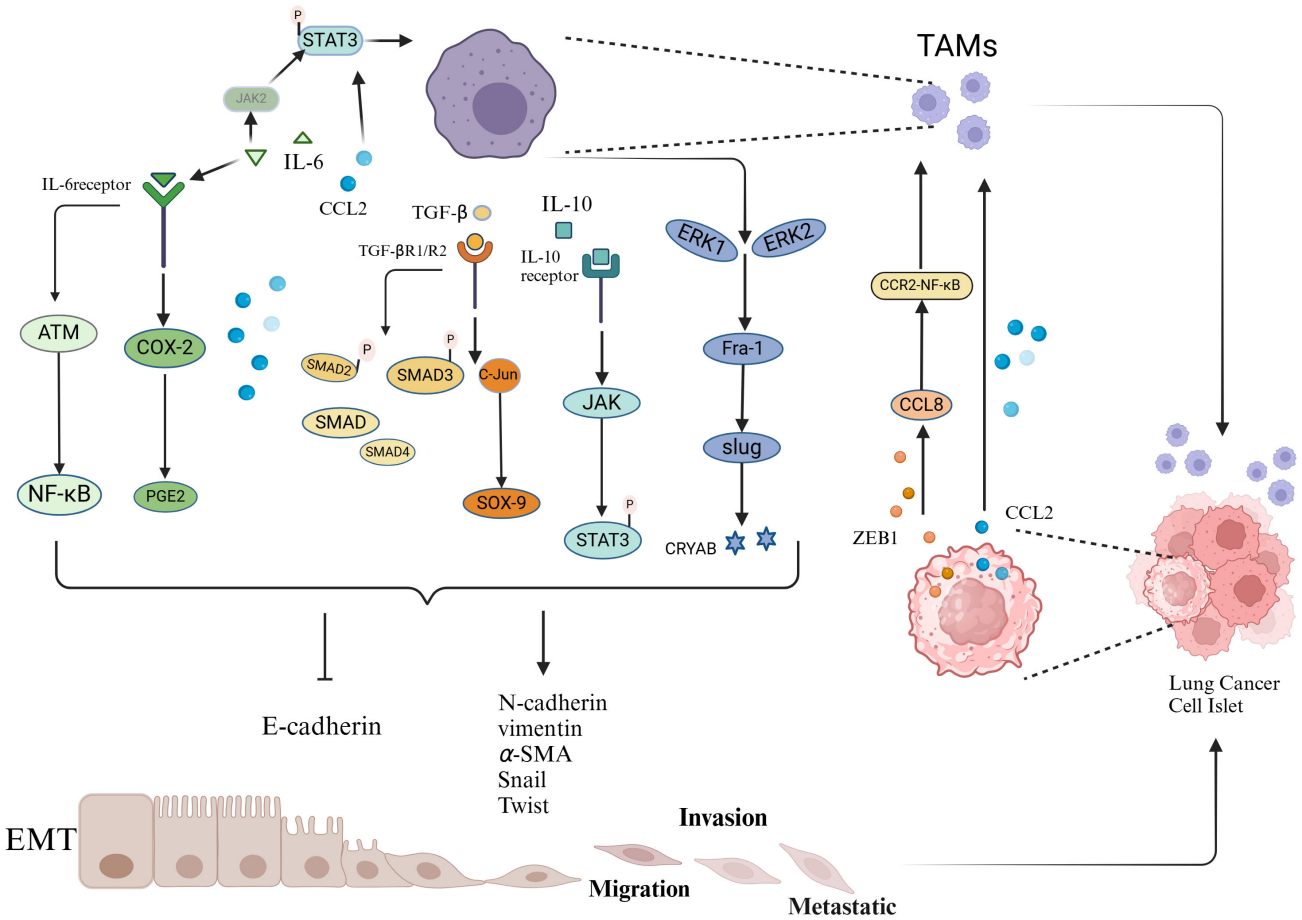
Schematic representation of the interaction between TAMs and EMT.

TAMs secrete various cytokines and growth factors that act on relevant signaling pathways, directly or indirectly participating in the initiation, maintenance, and progression of EMT in lung cancer, significantly promoting the loss of cell polarity and weakening intercellular adhesion, thereby disrupting the stability of intercellular connections (**Figure 1**). This process provides an important biological basis for the migration and invasion of tumor cells, serving as a key step in the malignant progression and metastasis of lung cancer.

TAMs promote the EMT of tumor cells by secreting various factors, including TGF-β, IL-6, and CCL2. These factors act on intracellular signaling pathways, including COX-2/PGE2, STAT3-C/EBPβ, ATM/NF-κB, TGF-β-SMAD, SMAD/ZEB, ERK1/2/Fra-1/Slug, and JAK1-STAT3. These pathways suppress the expression of epithelial markers while promoting the expression of mesenchymal markers, thereby inducing EMT in tumor cells. Lung cancer cells undergoing EMT secrete increased levels of chemokines and transcription factors, such as the chemokine CCL2 and the transcription factor ZEB1, further enhancing the recruitment and polarization of TAMs.

(1) IL-6

Interleukin-6 (IL-6) is a multifunctional cytokine that plays a significant role in regulating the immune system (68). Research by Dehai et al. (69) found that THP-1-derived macrophages promote the invasiveness and EMT process of lung adenocarcinoma cells by secreting IL-6. In another study, IL-6 was shown to promote the nuclear translocation of β-catenin via the COX-2/PGE2 pathway, thereby inducing EMT and enhancing tumor cell invasion (70). Furthermore, a study by Hu et al. (71) reported that TAMs promote IL-6 expression and activate the EMT pathway by forming an IL6-STAT3-C/EBPβ-IL6 positive feedback loop. Notably, IL-6 is also associated with chemotherapy resistance and prognosis in lung cancer patients; studies have shown that IL-6 can activate and enhance chemotherapy resistance in lung cancer by activating the ATM/NF-κB pathway (72). Additionally, high levels of IL-6 in plasma are regarded as important markers for poor prognosis in chemotherapy patients (73).

(2) TGF-β and related signaling pathways

Transforming growth factor-beta (TGF-β) is a prototypical member of a structurally and functionally related family of proteins that regulate cell proliferation, migration, and the differentiation of a variety of cell types (74). In the tumor microenvironment, TGF-β has a dual role in tumor progression. In the early stages of cancer, it acts as an effective tumor suppressor by inhibiting the cell cycle and promoting apoptosis, thereby suppressing tumor initiation and progression. However, in the middle to late stages of cancer, tumor cells may develop resistance to TGF-β or be reprogrammed by it (75), transforming TGF-β into a tumor promoter that induces epithelial–mesenchymal transition (EMT) and enhances the invasive and metastatic capabilities of tumor cells, as well as their resistance to chemotherapy. Furthermore, it supports cancer growth and progression by activating tumor angiogenesis and cancer-associated fibroblasts, as well as enabling tumors to evade immune responses (74, 76).

TGF-β is a key factor released by TAMs and has been shown to significantly promote the transition of epithelial cells to mesenchymal cells by activating various intrinsic pathways, such as the AKT, SMAD, and β-catenin pathways, thereby increasing cell migration and invasion (77–81). In the lung cancer microenvironment, TAMs can promote EMT in tumor cells by releasing TGF-β, which acts on signaling pathways such as the Smad/ZEB and C-jun/SMAD3 pathways, thereby increasing the metastatic potential and proliferation ability of tumors (82, 83). Research by J. A et al. (84) further revealed that B7-H4-expressing macrophages may regulate the EMT process by secreting TGF-β1, thereby promoting pleural metastasis in lung cancer. Additionally, a study by Bonde et al. (64) reached similar conclusions, indicating that TAMs induce EMT in cancer cells within tumors through TGF-β signaling and the β-catenin pathway.

(3) CCL2

CCL2 is a key chemokine that regulates the migration of monocytes/macrophages into the TME (85, 86). In lung cancer tissues, CCL2 secreted by TAMs promotes EMT through a dual regulatory mechanism: on one hand, it significantly downregulates the expression of the epithelial marker E-cadherin, while on the other, it simultaneously upregulates the expression levels of the mesenchymal marker vimentin, as well as matrix metalloproteinases MMP-2 and MMP-9, thereby enhancing the invasion and migration capabilities of NSCLC cells (87). Notably, CCL2 and IL-6 exhibit a significant synergistic effect in EMT induction. They mutually induce each other to activate STAT3 phosphorylation. In turn, activated STAT3 can regulate the expression of IL-6 and CCL2, forming a positive feedback loop that not only amplifies STAT3 signaling but also drives a cascade reaction in the EMT process. Additionally, CCL2 can significantly enhance IL-6-induced EMT by upregulating the expression of the transcription factor Twist (88).

(4) Other Pathways

In addition to the aforementioned classical cytokine pathways, several studies have revealed non-classical mechanisms by which TAMs regulate EMT in lung cancer: Li et al. (38) found that exosomal miR-155/miR-196a-5p can promote EMT in NSCLC cells through epigenetic modifications. Cao et al. (89) identified the IL-10/STAT3 phosphorylation cascade as a critical activator of EMT. Guo et al. (90) revealed that M2 macrophages enhance EMT by upregulating CRYAB expression and activating the ERK1/2/Fra-1/Slug signaling axis.

### 4.2 Feedback regulation of TAM function via the EMT process in lung cancer

EMT, while promoting tumor invasion and metastasis, is often accompanied by the attraction and infiltration of TAMs (91, 92). After undergoing EMT, tumor cells typically release increased levels of chemokines (such as CCL2), further promoting the recruitment and polarization of TAMs. This interaction forms a positive feedback loop that collectively drives tumor progression (**Figure 1**). Currently, the regulatory mechanisms by which EMT influences TAMs primarily involve the following pathways:

(1) TWIST1/CCL2-Mediated Macrophage Recruitment and M2 Polarization

TWIST1 is a key regulatory factor in the interplay between TAMs and EMT. On one hand, TWIST1 directly participates in the EMT process by regulating E-cadherin and other related proteins, thereby promoting EMT in lung cancer cells. On the other hand, activated TWIST1 upregulates the expression of CCL2, which acts as a chemokine to attract macrophages and induce their polarization toward the M2 phenotype. The polarized M2 macrophages, in turn, promote EMT in lung cancer cells, forming a positive feedback loop that continuously drives tumor progression and metastasis (93). Additionally, Wang et al. (42) found that elevated CtBP1 protein expression can induce EMT in NSCLC cells and regulate the activation of the NF-κB signaling pathway, leading to increased CCL2 secretion, which in turn promotes TAM recruitment and polarization.

(2) ZEB1 Promotes TAM Accumulation and Infiltration in the Hypoxic TME

Hypoxia is a critical microenvironmental stressor that regulates various potent immunosuppressive phenomena associated with tumor progression (94). Hypoxia triggers EMT in various cancers, including breast, prostate, and oral cancers (95). The accumulation of TAMs in the hypoxic TME is closely associated with malignant tumor progression. Moreover, hypoxic regions exhibit significant spatial overlap with EMT invasion fronts and TAM distribution (95, 96). ZEB1 is a key transcription factor in EMT, endowing cancer cells with an invasive, mesenchymal-like phenotype and serving as a predictor of poor clinical prognosis in most cancers. In the hypoxic TME, ZEB1 promotes macrophage infiltration by activating the transcription of CCL8, which subsequently attracts macrophages via the CCR2-NF-κB pathway, enhancing TAM accumulation in the tumor microenvironment (97).

In addition to the aforementioned factors, various inflammatory cytokines induced during the EMT process, such as the pro-inflammatory and immunoregulatory TNF-α (98), neutrophil-recruiting GROs (99), and angiogenesis-promoting IL-8 (100), also play crucial roles in the interaction between EMT and TAMs. These factors may collectively contribute to creating a favorable environment for tumor development.

The interaction between TAMs and EMT is also significant in other types of cancer. In pancreatic ductal adenocarcinoma, Xiong et al. (101) demonstrated that TAMs drive EMT by activating the Snail transcription factor via the TGF-β/Smad2/3/4 signaling axis, thereby promoting stromal invasion and tumor microenvironment remodeling. In hepatocellular carcinoma, Zhang et al. (102) discovered a HIF-1α/IL-1β signaling loop between cancer cells and TAMs in the hypoxic microenvironment, leading to epithelial-mesenchymal transition and metastasis. In rectal cancer, Zheng et al. (103) found that the long non-coding RNA LINC00543 enhances EMT through the pre-miR-506-3p/FOXQ1 axis, leading to the upregulation of CCL2, which in turn promotes CCL2-mediated macrophage recruitment and M2-like polarization. Further research by Wei et al. (104) confirmed that TAMs can enhance EMT progression via the STAT3/miR-506-3p/FoxQ1 pathway, leading to increased CCL2 production, which facilitates macrophage recruitment. This establishes an “EMT-CCL2-TAM” positive feedback loop that drives rectal cancer progression. In breast cancer, Su et al. (105) found that EMT-processed cancer cells secrete GM-CSF, which activates macrophages into a TAM-like phenotype. The activated macrophages, in turn, secrete CCL18, inducing EMT in cancer cells and establishing a positive feedback loop. This loop is crucial for promoting breast cancer cell metastasis and is associated with poor prognosis in breast cancer patients.

In summary, the interaction between TAMs and EMT creates a positive feedback mechanism that not only enhances the migratory capacity of tumor cells but also leads to sustained immune suppression within the tumor microenvironment, facilitating tumor cell escape from host immune surveillance and clearance.

## 5 Therapeutic strategies targeting TAMs and EMT

Therapeutic strategies targeting TAMs and EMT are being progressively developed, showing promising preclinical prospects. By inhibiting the recruitment or polarization of TAMs, their supportive role in tumors can be diminished, thereby enhancing the efficacy of conventional treatments such as chemotherapy and radiotherapy. Additionally, blocking the EMT process to reduce the migration, invasion, and drug resistance of tumor cells is also considered a highly promising strategy for lung cancer treatment. Here, we summarize the current therapeutic strategies targeting TAMs and EMT as key points (**Table 2**) and outline the progress of lung cancer clinical trials related to TAMs and EMT (**Table 3**).

**Table 2:** Therapeutic approaches targeting TAMs and EMT in lung cancer.

Pharmacological Agent	*In vivo*/*In vitro*	Mechanism	Approach	Reference
Targeting TAMs				
Imatinib	*In vivo* and *in vitro*	Inhibition of STAT6 phosphorylation and nuclear translocation	Inhibits M2-like polarization of TAMs	(106)
Resveratrol	*In vivo* and *in vitro*	Reduced STAT3 activity and p-STAT3 expression	Inhibits M2-like polarization of TAMs	(107)
Paeoniflorin	*In vivo* and *in vitro*	unknown	Decrease in the number of M2 macrophages	(108)
Astragaloside IV	*In vitro*	Reduces p-AMPK levels	Inhibits M2-like polarization of TAMs	(109)
Puerarin	*In vivo* and *in vitro*	Inactivate MEK/ERK 1/2 pathway	Inhibits M2-like polarization of TAMs	(110)
Gefitinib	*In vivo* and *in vitro*	Inhibits STAT6 phosphorylation	Inhibits M2-like polarization of TAMs	(111)
lapatinib	*In vivo* and *in vitro*	Inhibition of IL-13-triggered STAT6 phosphorylation	Inhibits M2-like polarization of TAMs	(112)
Anagliptin	*In vivo* and *in vitro*	Inhibition of M-CSF -induced NOX1 and NOX2 expression suppresses reactive oxygen species production in bone marrow monocytes, reduces late ERK signaling pathway activation, and suppresses monocyte-macrophage differentiation	Inhibits macrophage differentiation and M2 macrophage polarization	(113)
Oyster enzymatic hydrolysate (OEH)	*In vivo*	unknown	Reducing the number of TAMs	(114)
JWH-015	*In vivo* and *in vitro*	Downregulation of EGFR pathway	Inhibition of TAMs recruitment and EMT	(115)
Ginsenoside Re	*In vivo* and *in vitro*	Inhibition of AMPKα1/STING positive feedback loop formation	Inhibition of M2 type polarization of TAMs	(40)
13-Methyl-palmatrubine	*in vitro*	Inhibition of PI3K/AKT and JAK2/STAT3 signaling pathway activation	Shift the polarization of the TAMs from M2 to M1	(116)
β-elemene	*In vitro*	unknown	Regulates macrophage polarization from M2 to M1	(117)
Hydroxychloroquine	*In vivo* and *in vitro*	unknown	promote the transition of M2-TAMs into M1-like macrophages	(118)
Betulinic acid	*In vivo* and *in vitro*	Inhibits mTOR signaling pathway	Repolarization of tumor-associated macrophages increases the ratio of M1/M2 macrophages in tumor tissue	(119)
Erastin	*In vivo* and *in vitro*	Inhibition of xCT-mediated AKT/STAT6 signaling	Reduced M2-type polarization of TAMs and boosts responses to immune checkpoint blockade	(120)
Targeting EMT				
Harmine	*In vivo* and *in vitro*	Suppression of the TWIST1 gene	Overcoming EMT-mediated resistance to EGFR TKIs	(121)
TP-0903	*In vivo* and *in vitro*	Suppression of AXL	Reverses EMT process	(122)
BGB324	*In vivo* and *in vitro*	Suppression of AXL	Blocking the EMT process to overcome EMT-associated drug resistancein cancer	(123)
Bufalin	*In vitro*	Downregulates TGF-β receptor	Suppression of EMT and migration	(124)
Methionine enkephalin (MENK)	*In vivo* and *in vitro*	Interaction with opioid growth factor receptors	Inhibition of the EMT process	(125)

**Table 3:** Clinical trials related to TAMs and EMT in lung cancer therapy.

Strategy	Cancer types	Clinical phase	Status/out-comes	Clinical identifier	Location
68GaNOTA-Anti-MMR-VHH2	NSCLC	II	Recruiting	NCT05933239	Belgium
CT-0508 combined with Pembrolizumab	Lung Cancer	I	Active, not recruiting	NCT04660929	Unite States
DRibble vaccine combined with cyclophosphamide, +/-GM-CSF/imiquimod	NSCLC	II	Completed	NCT01909752	Unite States
GM-CSF Plus Maintenance Pembrolizumab +/- Pemetrexed	NSCLC	II	Recruiting	NCT04856176	Unite States
Recombinant Human Vascular Endothelial Inhibitor (Endo) in Combination With Brag	NSCLC	II	Recruiting	NCT06047860	CHINA
TQB2928 in Combination With a Third-Generation EGFR TKI	NSCLC	I	Not yet recruiting	NCT06585059	CHINA
Amivantamab With Tyrosine Kinase Inhibitors (TKI)	NSCLC	I&II	Recruiting	NCT05845671	Unite States
Amivantamab, Lazertinib and Pemetrexed	NSCLC	II	Recruiting	NCT05299125	Brazil

∗)The data source from https://www.clinicaltrials.gov and the latest update is November 4, 2024.

### 5.1 Therapeutic strategies targeting TAMs

In the lung cancer microenvironment, TAMs constitute a major component of immune cells. They not only act as immunosuppressive cells, enabling lung cancer cells to escape immune surveillance but also directly promote cancer cell proliferation, survival, invasion, and metastasis (126). Therefore, the precise regulation and development of therapeutic strategies targeting TAMs have become important research directions in the field of lung cancer treatment.

(1) Modulating the number of TAMs

As mentioned earlier, M2-type TAMs dominate the lung cancer microenvironment, and their high infiltration at tumor sites is a crucial factor in tumor progression. Therefore, reducing the density of TAMs, particularly the number of M2-type TAMs, can effectively inhibit lung cancer progression. Blocking various chemokines produced by tumor and stromal cells, such as monocyte chemoattractant protein-1 (MCP-1), prostaglandin E2 (PGE2), colony-stimulating factor 1, and CCN3, can inhibit the recruitment of TAMs to tumor sites, thereby suppressing tumor progression and preventing metastasis (127, 128). Several drugs, such as ginsenoside (40), imatinib (106), resveratrol (107), paeoniflorin (108), astragaloside IV (109), puerarin (110), gefitinib (111), apatinib (112), and anagliptin (113), have been shown to inhibit M2 polarization of macrophages and reduce TAM infiltration, thereby suppressing lung cancer progression. Additionally, two studies indicated that oyster hydrolysate (OEH) and cannabinoid receptor-2 agonists could reduce the recruitment of macrophages to tumor sites and inhibit M2-type macrophage-induced EMT, thereby decreasing the migration and invasion capabilities of lung cancer cells (114, 115). On the other hand, increasing the number of M1-type TAMs is also considered an effective approach to suppress tumors. Liu et al. (129) found that the overexpression of the transcription factor TCF21 promotes the polarization of TAMs to M1 macrophages and enhances the impact of macrophages on T-cell activity, thereby strengthening the body’s antitumor immune response.

(2) Altering the function of TAMs

Given that TAMs exhibit significant plasticity and can polarize into different phenotypes in response to various stimuli in the tumor microenvironment, “reprogramming” TAMs to adopt an antitumor phenotype represents a promising therapeutic strategy. A series of drugs, such as 13-methyl-palmatrubine (13MP) derived from the flower of Crotalaria juncea (116), β-caryophyllene (117), hydroxychloroquine (118), and birch bark acid (119), have been found to regulate the polarization state of TAMs by modulating signaling pathways such as the AMPKα1/STING, mTOR, PI3K/AKT, and JAK/STAT3 pathways, facilitating the transition from the tumor-promoting M2 phenotype to the antitumor M1 phenotype and thereby inhibiting lung cancer growth and progression. Additionally, Lee et al. (130) found that inhibiting the Wnt/β-catenin signaling pathway can also reprogram TAMs into an M1-like phenotype that suppresses tumors. Lin et al. (131) achieved combined administration of osimertinib and panobinostat (Pan) via a liposome codelivery system modified with lactoferrin; this strategy not only reversed EMT-related resistance by repolarizing TAMs from the M2 phenotype to the M1 phenotype but also inhibited tumor metabolism and angiogenesis, providing a new therapeutic approach to overcoming resistance in NSCLC. Notably, the application of nanotechnology, such as ultrasound-mediated PLGA-PEI nanobubbles carrying STAT6 siRNA and intraperitoneal injection of HA-PEI nanoparticles, has also demonstrated the ability to repolarize TAMs from the M2 phenotype to the M1 phenotype, thereby inhibiting the progression of non-small cell lung cancer (132, 133).

(3) TAMs and immunotherapy in lung cancer

TAMs play a crucial role in immunotherapy for lung cancer. They inhibit phagocytosis and tumor immunity by expressing PD-1, and blocking PD-1/PD-L1 *in vivo* can enhance macrophage phagocytosis and reduce tumor growth. Monoclonal antibodies that block PD-1/PD-L1 have demonstrated significant clinical efficacy in various cancer patients, including those with non-small cell lung cancer (NSCLC) (134). In NSCLC, MARCO-expressing TAMs exhibit an M2 phenotype that promotes tumor growth, and this phenotype is positively correlated with immune response pathways (135). The polarization state of TAMs can affect the activity of NF-κB, which participates in immune evasion by regulating PD-L1 expression in tumor cells. Thus, inhibiting the NF-κB signaling pathway may help reduce PD-L1 expression and enhance the efficacy of immunotherapy (136). Furthermore, xCT derived from TAMs is closely associated with poor prognosis in lung cancer patients. xCT deficiency downregulates the AKT/STAT6 signaling pathway, inhibits M2 polarization of macrophages, increases T-cell infiltration, and activates inflammatory and immune responses. The xCT inhibitor erastin enhances sensitivity to PD-L1 and effectively suppresses lung cancer progression (120). Additionally, several other molecules and signaling pathways that target TAMs have been identified in lung cancer, such as TCF21 (129), ADPGK-AS1 (137), TLR4 (138) and JMJD6/STAT3/IL-10 (139), which are also considered potential targets for immunotherapy. Notably, the recently introduced chimeric antigen receptor macrophage (CAR-M) therapy has opened new possibilities for the immunotherapy of solid tumors (140). Although CAR-M has not yet been reported for the treatment of lung cancer, several CAR-M therapies, such as CT-0508 (NCT04660929) and MCY-M11 (NCT03608618), have been approved by the U.S. Food and Drug Administration (FDA) to enter clinical trials for the treatment of recurrent or metastatic solid tumors overexpressing HER2 as well as recurrent/refractory ovarian cancer and peritoneal mesothelioma (141, 142). With continuous advancements in gene editing technologies, synthetic biology, and biomaterials science, CAR-M therapy is expected to become a crucial component of cancer immunotherapy, providing a powerful tool for overcoming solid tumors, including lung cancer.

### 5.2 Therapeutic strategies targeting EMT

The EMT process plays a crucial role in the metastasis, immune evasion, and chemotherapy resistance of lung cancer. Therefore, therapeutic strategies targeting EMT are particularly important.

(1) EGFR-AKT:

Epidermal growth factor receptor (EGFR)-mutant tumors have become key targets in the study of lung cancer resistance since their discovery in 2004. Currently, the third-generation EGFR inhibitor osimertinib is widely used in first-line treatment (143). However, in recent years, several previously uncommon acquired resistance mechanisms have emerged in lung cancer patients treated with osimertinib, with increasing frequency. These mechanisms include acquired EGFR mutations (e.g., C797S), amplification of MET and HER2, and small-cell transformation (144–146). Notably, epithelial–mesenchymal transition (EMT) transcription factors such as ZEB1, Slug, and TWIST1 have been identified as drivers of EGFR TKI resistance mediated by EMT, suggesting that these transcription factors could be potential targets for treating EMT-related resistance in lung cancer (121, 147–149).

(2) Regulation of microRNAs and circRNAs:

Research on EMT-related microRNAs and circRNAs has provided new therapeutic approaches and strategies for lung cancer patients. Specifically, studies have shown that in cancer cells undergoing Snail-induced epithelial–mesenchymal transition (EMT), tumor cells can deliver miR-21 via exosomes to inhibit the activity of the NLRP3 inflammasome in TAMs, thereby enhancing the resistance of tumor cells (150). Another study (151) revealed that miR-138-5p inhibits the epithelial–mesenchymal transition, growth, and metastasis of lung adenocarcinoma cells by targeting ZEB2. These findings suggest that modulating specific microRNAs could be an effective strategy for targeting EMT. Additionally, Jiang et al. (152) discussed the roles of EMT-inducing and EMT-suppressing circRNAs in lung cancer. They reported that EMT-inducing circRNAs primarily promote EMT-mediated metastasis by affecting key members of EMT-related signaling pathways in NSCLC (e.g., Wnt/β-catenin), whereas EMT-suppressing circRNAs play a significant role in inhibiting EMT-mediated metastasis in NSCLC by acting as sponges for microRNAs, influencing the expression of EMT transcription factors (EMT-TFs), EMT-related signaling, and EMT markers.

(3) Targeting the TAM Family Receptor Tyrosine Kinase AXL:

AXL is a receptor tyrosine kinase of the TAM family that has become an important factor influencing the resistance of NSCLC and other cancers to chemotherapy, radiotherapy, and targeted therapy because of its key role in mediating EMT and immune evasion (153, 154). Studies have indicated that AXL expression is significantly upregulated in EGFR-mutant non-small cell lung cancer cells that have developed resistance and that its degradation rate is inhibited. Therefore, regulating the degradation rate of AXL is expected to be a new strategy to overcome gefitinib resistance (155). Several studies have shown that Axl inhibitors can increase the sensitivity of tumors to chemotherapy and radiotherapy and may also help overcome tumor immune evasion (122, 123, 156, 157). Furthermore, the combination of AXL with ATR inhibitors has been shown to be an effective strategy for treating lung cancer. This approach not only increases the sensitivity of non-small cell lung cancer (NSCLC) cells to ATR inhibitors but can also be used to treat SLFN11-low tumors resistant to platinum-based and PARP inhibitors while inhibiting the progression of small cell lung cancer (SCLC) by modulating tumor-associated macrophages (TAMs) and the epithelial–mesenchymal transition (EMT) (158, 159).

In addition to the aforementioned pathways, current strategies targeting the EMT process for lung cancer treatment involve various molecules and mechanisms that play significant roles in inhibiting epithelial–mesenchymal transition (EMT) in lung cancer, opening new perspectives and providing new ideas for its treatment. Among these factors, TGF-β, a key factor in inducing EMT, is also regarded as an important target for inhibiting the EMT process in lung cancer. For example, research has indicated that bufalin can inhibit the EMT process induced by TGF-β in human lung cancer A549 cells by downregulating the expression of TGF-β receptors (124). Additionally, Zhang et al. (125) reported that methionine enkephalin (MENK) can inhibit the growth, migration, invasion, and EMT of lung cancer cells by interacting with opioid growth factor receptors, thereby combating lung cancer. Research by Kim et al. (160) indicated that apoptotic cancer cells treated with ultraviolet irradiation can secrete PTEN (phosphatase and tensin homolog) and PPARγ (peroxisome proliferator-activated receptor gamma) ligands, which inhibit EMT and metastasis in lung cancer cells.

## 6 Conclusion and outlook

Tumor-associated macrophages (TAMs) play crucial roles in the process of epithelial–mesenchymal transition (EMT) in lung cancer. TAMs promote EMT in tumor cells by releasing cytokines and growth factors, significantly enhancing the migration and invasion capabilities of tumor cells. Moreover, tumor cells that have undergone EMT alter the microenvironment, impacting the function of TAMs and thereby creating a positive feedback loop. This complex interaction not only deepens our understanding of the biology of lung cancer but also provides new targets for the development of therapeutic strategies.

With further research into the mechanisms of TAMs and EMT, we hope to discover more effective biomarkers and explore treatment strategies based on these mechanisms. Future research should focus on elucidating the specific roles of TAMs and EMT in different tumor microenvironments, as well as how to translate these findings into clinical applications to increase treatment efficacy and improve patient outcomes.

In summary, TAMs play an important role in the EMT process in lung cancer, and a deeper understanding of their interactions will help us better comprehend the complexities of lung cancer and provide more effective treatment options for patients.
